# Melatonin Influences Structural Plasticity in the Axons of Granule Cells in the Dentate Gyrus of Balb/C Mice

**DOI:** 10.3390/ijms20010073

**Published:** 2018-12-25

**Authors:** Gerardo Bernabé Ramírez-Rodríguez, Sandra Olvera-Hernández, Nelly Maritza Vega-Rivera, Leonardo Ortiz-López

**Affiliations:** 1 Laboratorio de Neurogenesis, Subidrección de Investigaciones Clínicas, Instituto Nacional de Psiquiatría “Ramón de la Fuente Muñiz”, Calzada México-Xochimilco 101, Col. San Lorenzo Huipulco, Tlalpan, México City C.P. 14370, México; olverhs@yahoo.com.mx (S.O.-H.); leosan@imp.edu.mx (L.O.-L.); 2 Laboratorio de Neuropsicofarmacología, Dirección de Neurociencias, Instituto Nacional de Psiquiatría “Ramón de la Fuente Muñiz”, Calzada México-Xochimilco 101, Col. San Lorenzo Huipulco, Tlalpan, México City C.P. 14370, México; nmvega@imp.edu.mx

**Keywords:** melatonin, hippocampus, aging, mossy fibers, calbindin, adult hippocampal neurogenesis

## Abstract

Melatonin, the main product synthesized by the pineal gland, acts as a regulator of the generation of new neurons in the dentate gyrus (DG). Newborn neurons buffer the deleterious effects of stress and are involved in learning and memory processes. Furthermore, melatonin, through the regulation of the cytoskeleton, favors dendrite maturation of newborn neurons. Moreover, newborn neurons send their axons via the mossy fiber tract to Cornu Ammonis 3 (CA3) region to form synapses with pyramidal neurons. Thus, axons of newborn cells contribute to the mossy fiber projection and their plasticity correlates with better performance in several behavioral tasks. Thus, in this study, we analyzed the impact of exogenous melatonin (8 mg/kg) administered daily for one- or six-months on the structural plasticity of infrapyramidal- and suprapyramidal mossy fiber projection of granule cells in the DG in male Balb/C mice. We analyzed the mossy fiber projection through the staining of calbindin, that is a calcium-binding protein localized in dendrites and axons. We first found an increase in the number of calbindin-positive cells in the granular cell layer in the DG (11%, 33%) after treatment. Futhermore, we found an increase in the volume of suprapyramidal (>135%, 59%) and infrapyramidal (>128%, 36%) mossy fiber projection of granule neurons in the DG after treatment. We also found an increase in the volume of CA3 region (>146%, 33%) after treatment, suggesting that melatonin modulates the structural plasticity of the mossy fiber projection to establish functional synapses in the hippocampus. Together, the data suggest that, in addition to the previously reported effects of melatonin on the generation of new neurons and its antidepressant like effects, melatonin also modulates the structural plasticity of axons in granule cells in the DG.

## 1. Introduction

Melatonin is synthesized and released by the pineal gland [1,2]. The indole acts through membrane receptors [1,2]. Melatonin shows antioxidant properties and interacts with intracellular proteins [3,4,5,6,7,8] including, but not limited to, calcium-binding proteins such as the alpha-isoform of protein kinase C (PKCα), and calmodulin (CaM) [3,4,5,6,9,10] presumably to induce changes in cytoskeleton organization [6,9,11,12]. In this regard, it is possible that, through the activation of intracellular proteins, melatonin promotes the accumulation of microtubule-associated protein 2 (MAP2) of cells in the hilar zone in the dentate gyrus (DG) in rodents [13,14]. It is also known that melatonin maintains calcium-binding calretinin-positive neurons in the DG during aging [15]. This protein is essential for murine hippocampal neurogenesis [16,17] and its expression declines during aging [18,19,20]. Interestingly, the generation of new neurons in the DG decline during aging [21]. However, melatonin promotes cell survival, enhances dendrite maturation of new neurons, and the indole delays the decline of adult hippocampal neurogenesis [13,14,15,22,23,24,25]. In this regard, it has been suggested that some neuropsychiatric disorders occur with alterations of the hippocampal neurogenic process [26]. Reduced plasma melatonin levels are also altered in several neuropsychiatric disorders including, but not limited to, Alzheimer’s disease, schizophrenia, obsessive-compulsive disorder. This process also occurs as we age [21,27,28].

Moreover, in the course of adult dentate gyrus hippocampal neurogenesis, newborn neurons send their axons via the mossy fiber tract to the location of Cornu Ammonis 3 (CA3), in which they form functional synapses with pyramidal neurons (i.e., [29]). The mossy fiber subfields comprise the suprapyramidal and the infrapyramidal mossy fiber projections. Interestingly, the structural plasticity of the infrapyramidal mossy fiber projection is influenced by increased neurogenesis and the axons of newborn neurons contribute to form the infrapyramidal projection [30]. Moreover, it has been reported that the infrapyramidal projection correlates with performance in several behavioral tests such as swimming, navigation, and spatial learning [31,32,33].

Considering the above, in addition to the modulation of a generation of new neurons and the dendrite complexity of doublecortin-associated cells in the DG [13,14,15,22,23,24,25], we hypothesized that melatonin could regulate the structural plasticity and the distribution of mossy fibers which are formed by both the axons of newborn neurons, and mature granule cells in the DG of adult mice [30].

In our study, we demonstrated that chronic administration of melatonin increases the number of calbindin-positive cells and affects the structural plasticity of both suprapyramidal and infrapyramidal mossy fiber projections also identified with calbindin, the main calcium-buffering protein in mossy fibers [34], after one- or six-months of treatment in male Balb/C mice. Thus, our data suggest that, as part of the regulation caused by melatonin in the dentate gyrus through the generation of newborn neurons [13,14,15,22,23,24,25], the indole also contributes to the modifications of structural plasticity of the mossy fibers in the dentate gyrus, an effect that may also have implications on the formation of a connection between the dentate gyrus and CA3 [30].

## 2. Results

### 2.1. Distribution of Calbindin in the Dentate Gyrus in Male Balb/c Mice

**Figure 1:**
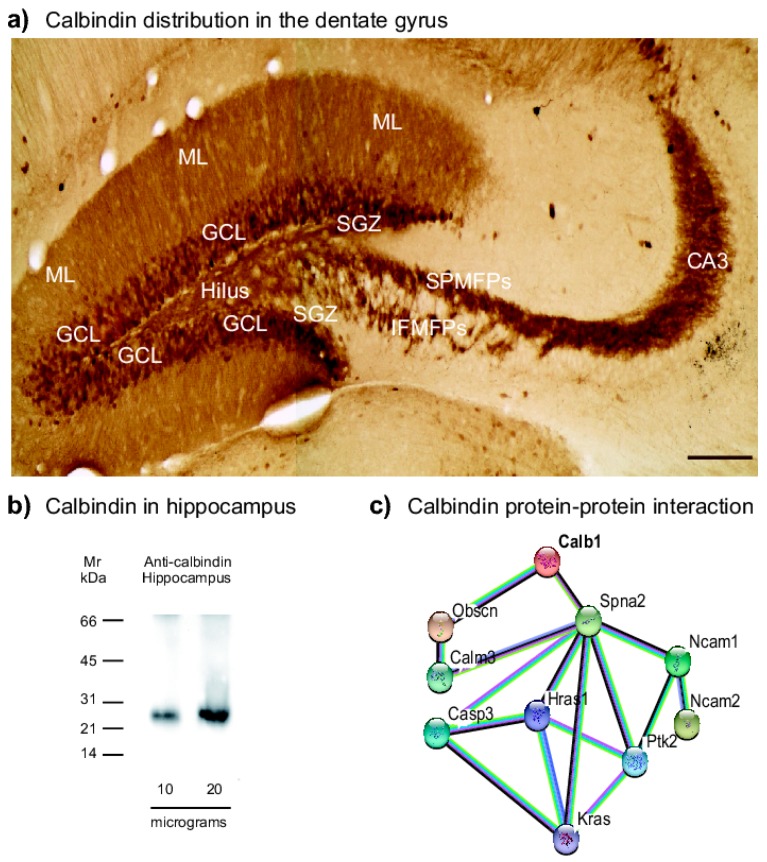
Calbindin distribution along the dentate gyrus. (**a**) Mossy fiber projections were visualized with an anti-calbindin antibody. Bright fields also show the granule cell layer (GCL), subgranular zone (SGZ), hilus (H), molecular layer (ML), suprapyramidal (SPMFPs), infrapyramidal-mossy fiber projections (IPMFPs), and Cornu Ammonis 3 (CA3), respectively. Scale bar in (**a**) = 150 µm. (**b**) Western blot analysis of whole hippocampus protein lysates to identify calbindin within a 28 kDa of molecular weight. (**c**) Functional protein association network obtained from String database (https://string-db.org) is shown. The map shows the interaction of calbindin (Calb1) with Spna2 (fodrin) and Obscn (obscurin) that establishes the interaction with Ras proteins (Hras1, Kras), protein tyrosine kinase (Ptk2), and neural cell adhesion molecule (Ncam).

Firstly, we studied calbindin distribution along the dentate gyrus (Figure 1a). Calbindin labels a well-stained mossy fiber projection reaching the CA3 region in the hippocampus [34,35]. Calbindin is located in axonal boutons and dendritic spines [34,35]. Moreover, calbindin is located at the granular cell layer (Figure 1a) [30,35]. The antibody used for immunohistochemistry but tested in whole protein extracts of hippocampus by immunoblotting, showed a protein band of ~28 kDa, which corresponded to the expected molecular weight of calbindin (Figure 1b). Moreover, functional protein association network obtained from the String database (https://string-db.org, accessed on 10 September 2018) indicates that calbindin is involved in biological process including the regulation of actin cytoskeleton organization (G0:0032956), the regulation of long-term neuronal synaptic plasticity (GO:0048169), the regulation of synapse structure or activity (GO:0050803) (protein-protein interaction (PPI) enrichment, *p* = 0.00093; Figure 1c). Thus, the PPI analysis indicated that calbindin is a protein involved in key biological process related to the structure of synapses, including axonal growth.

### 2.2. Melatonin Modulates Plasticity of Axons in Granule Cells in the Dentate Gyrus in Male Balb/c Mice

**Figure 2:**
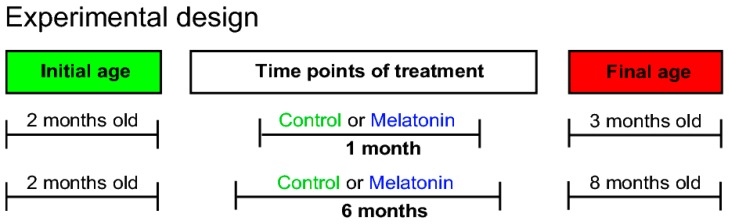
Experimental design. Melatonin was administered for one- or six-months. At the end of the treatment, the final ages of the mice were 3 or 8 months, respectively. Melatonin was prepared in a concentration to yield a dose of 8 mg/kg of body weight (b.w.) per day during the treatment. Five male Balb/C mice were included per group.

**Figure 3:**
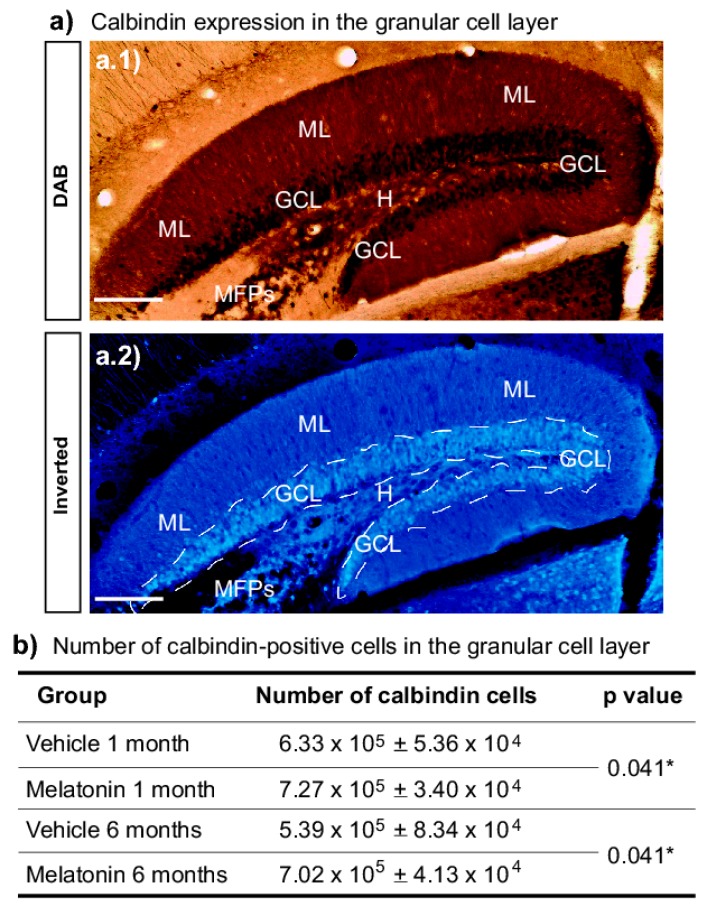
Number of calbindin-positive cells in the granular cell layer. (**a**) Representative pictures show calbindin expression in the granular cell layer (GCL). Images also show the molecular layer (ML), hilus (H), and the initial part of the mossy fiber projection (MFPs). The image in A.1 corresponds to a bright field and the image in A.2 is the inverted picture displayed in A.1. The dashed line indicates the GCL. Scale bar = 150 µm. (**b**) Calbindin-positive cells were quantified in the granular cell layer in the dentate gyrus. Two-way ANOVA yielded the following values: Factor A (treatment): F1,19 = 5.22, *p* = 0.041. Factor B (time): F1,19 = 1.13, non-significant (n.s.) Interaction AxB: F1,19 = 0.376, n.s. However, the significant main effect in the number of calbindin-positive cells was caused by treatment with melatonin (*).

Considering that previous studies have indicated that melatonin (8 mg/kg) induced neurogenesis occurs in male Balb/C mice [24], and that new neurons send their axons through the mossy fiber projection [30], we analyzed whether chronic administration of melatonin for one- or six-months (Figure 2) increased the number of calbindin-labeled cells in the granular cell layer in the dentate gyrus (Figure 3). We found that melatonin induced an increased number of calbindin-positive cells in the dentate gyrus after one- or six-months of treatment (11%, 33%; *p* = 0.041; respectively). Thus, the significant main effect in the number of calbindin-positive cells in the granular cell layer was caused by treatment with melatonin (F1,19 = 5.22, *p* = 0.041) (Figure 3b). Two-way ANOVA interaction between factor A (treatment) and factor B (time) yielded the following values: F1,19 = 0.376, non-significant (n.s).

**Figure 4:**
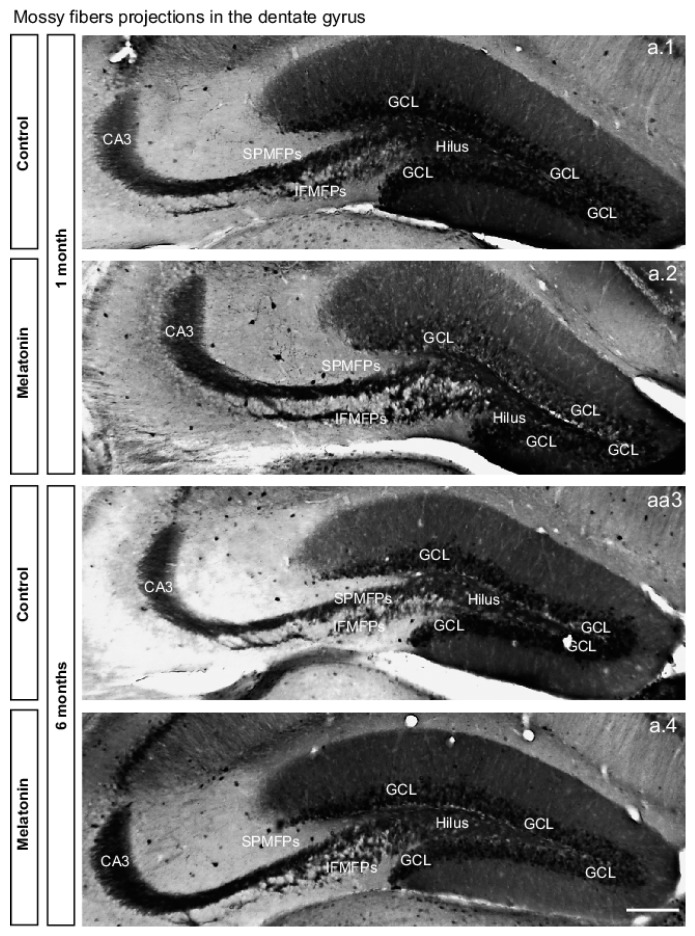
Expression of calbindin in the dentate gyrus after treatment with melatonin for one- or six-months in male Balb/C mice. (**a.1**–**a.4**) Representative images of coronal sections of mice treated with vehicle or melatonin (8 mg/kg, body weight; b.w;) sections are shown. Images of melatonin-treated mice show increased labeling of calbindin than that found in vehicle treated mice. Scale bar = 150 µm. The bright field also shows the granule cell layer (GCL), hilus (H), molecular layer (ML), suprapyramidal (SPMFPs) and infrapyramidal-mossy fiber projections (IPMFPs), and the Cornu Ammonis 3 (CA3) regions, respectively.

**Figure 5:**
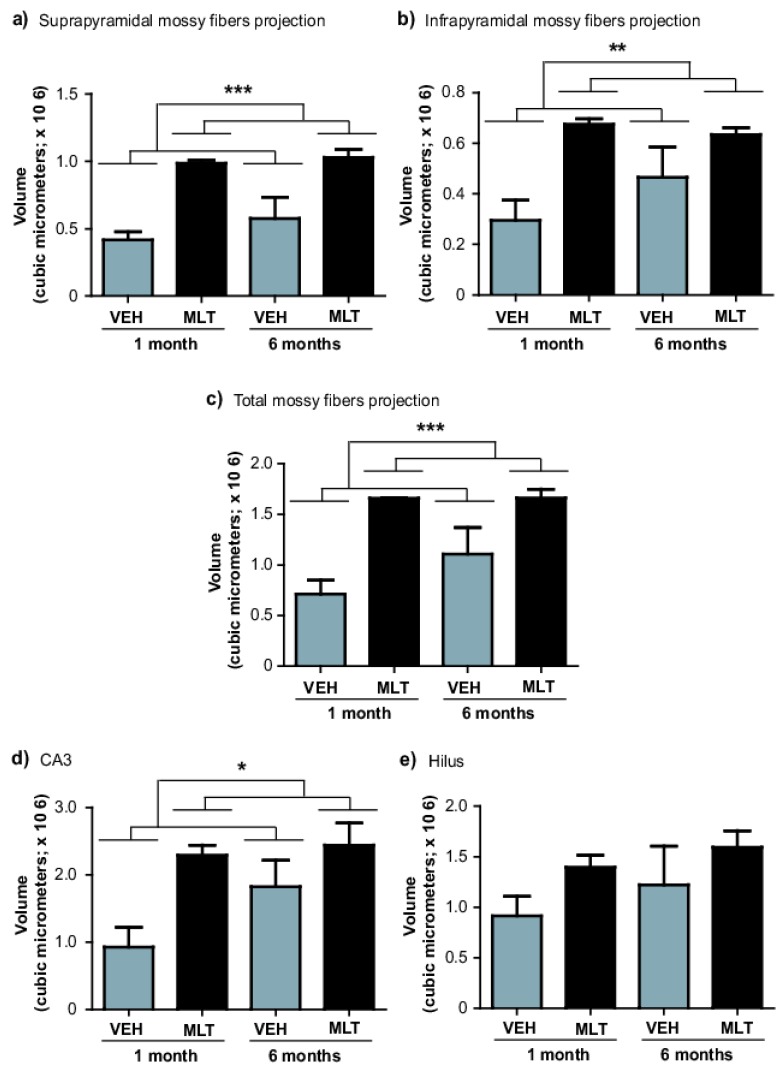
Volumes of mossy fiber tracts were increased by melatonin in male Balb/C mice. (**a**) Volume determination in the suprapyramidal mossy fiber projection indicates that melatonin significantly increased the volume in this subfield of the mossy fibers in the dentate gyrus. Error bars represent S.E.M. *** *p* < 0.0001. (**b**) Volume in the infrapyramidal mossy fiber projection indicates that one-month of treatment with melatonin significantly increased the volume in this subfield of the mossy fiber in the dentate gyrus compared to the vehicle (VEH). Error bars represent S.E.M. ** *p* = 0.002. (**c**) However, the total volume of the mossy fiber projection was significantly increased by melatonin in both time points. Error bars represent S.E.M. *** *p* < 0.0001. (**d**) Moreover, volume determination in the Cornu Ammonis 3 (CA3) region of the hippocampus shows that one-month of treatment with melatonin significantly increased the volume in this subfield of the hippocampus. Error bars represent S.E.M. * *p* = 0.028. (**e**) Finally, volume in the hilus was not significantly modified by melatonin. Error bars represent S.E.M. In all cases, a two-way ANOVA followed by Student Newman Keuls post hoc test was applied. Asterisks correspond to the main effect of treatment.

Thus, we analyzed whether chronic administration of melatonin for one- or six-months increased the volume of the mossy fibers (Figure 4 and Figure 5). Quantification of volume in the suprapyramidal mossy fiber projection showed significant differences among the groups after one- or six-months of treatment with melatonin (135%, 59%; *p* < 0.001; respectively) compared to the vehicle groups. Again, the significant main effect in the volume in the suprapyramidal mossy fiber projection was caused by treatment (F1,19 = 32.84, *p* < 0.001) (Figure 4 and Figure 5a. Two-way ANOVA interaction between factor A (treatment) and factor B (time) yielded the following values: F1,19 = 1.20, n.s.

Similarly, quantification of volume in the infrapyramidal mossy fiber projection revealed significant difference after one-month of treatment with melatonin (128%) compared to the vehicle group (*p* = 0.002). However, mice treated with melatonin during a six-month period showed an increased trend in the volume of the infrapyramidal mossy fiber projection (*p* = 0.12). However, the significant main effect in the volume in the infrapyramidal mossy fiber projection was caused by treatment (F1,19 = 13.91, *p* = 0.002) (Figure 4 and Figure 5b). Two-way ANOVA interaction between factor A (treatment) and factor B (time) yielded the following values: F1,19 = 2.05, n.s. However, the total volume of the mossy fiber projection (suprapyramidal plus infrapyramidal) was significantly increased in melatonin treated mice (132%, 49.90%, *p* < 0.001; respectively) compared to the vehicle groups. Again, the significant main effect in the total volume of the mossy fiber projection was caused by treatment with melatonin (F1,19 = 23.86, *p* < 0.001) (Figure 4 and Figure 5c). Two-way ANOVA interaction between factor A (treatment) and factor B (time) yielded the following values: F1,19 = 1.63, n.s.

Moreover, quantification of CA3 volume revealed significant difference after one-month of treatment with melatonin (146%) compared to the vehicle group (*p* = 0.028). Again, mice treated with melatonin for six-months showed an increased trend in the volume of CA3 (*p* = 0.17). However, the significant main effect in the CA3 volume was caused by treatment with melatonin (F1,19=5.45, *p* = 0.028) (Figure 4 and Figure 5d). Two-way ANOVA interaction between factor A (treatment) and factor B (time) yielded the following values: F1,19 = 2.98, n.s. Although, there are no differences among the groups were found regarding the volume of the hilus (Figure 4 and Figure 5e).

Finally, the results indicate that the effects of melatonin do not depend on the treatment duration. Also, the results indicate that the volumes of the mossy fiber projection (suprapyramidal and infrapyramidal) and CA3 region were regulated by melatonin.

## 3. Discussion

**Figure 6:**
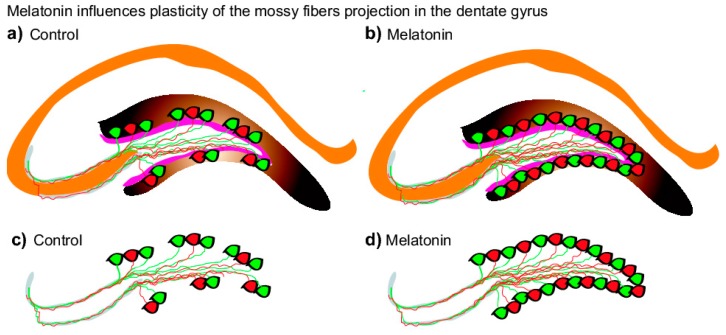
Schematic representation of the effects caused by melatonin on the volumes of mossy fiber projection. (**a**–**d**) After chronic administration of melatonin (8 mg/kg) for one- or six-months an increase in the mossy fiber projection was found in male Balb/C mice (**b**,**d**) compared to the vehicle treated group (**a**,**c**). Thus, the results suggest that melatonin plays a significant role in the plasticity of the mossy fibers projections that are also formed by axons of newborn- and mature-neurons in the dentate gyrus of the hippocampus.

In the present study, we analyzed the effects of melatonin on the structural plasticity in the mossy fiber projection in the dentate gyrus after chronic treatment with melatonin in male Balb/C mice for one- or six-months. Here, mossy fiber projection was identified by staining with calbindin, which is the main calcium-buffering protein in mossy fibers [34]. Melatonin increased the volumes of mossy fiber projection at different time points after treatment, suggesting that the indole contributes to the structural modifications of axons of granule cells in the dentate gyrus. Interestingly, melatonin increased the number of calbindin-positive cells in the dentate gyrus of Balb/C mice (Figure 6). This strain of mice was chosen because they show medium to low levels of baseline adult hippocampal neurogenesis but high relative numbers of surviving newborn cells in comparison to CD1 mice [36] and, at least in one study, were observed to have a large sensitivity to activity-induced regulation [37]. Also, our previous work has shown that melatonin exerts strong effects on cell proliferation, the survival of newborn neurons, and on the intermediate stages of neuronal development in the DG in Balb/C mice [24,25]. Interestingly, the effects of melatonin on neurogenesis could be strain-dependent. In this sense, exogenous melatonin positively increased cell proliferation, survival, and dendrite maturation in Balb/C- and C57Bl6-mice (i.e., [24,25]), which produce low levels of the indole [38,39]. However, exogenous melatonin did not favor cell proliferation or survival of newborn cells in the DG [40] of melatonin-proficient C3H/HeN mice [39]. Therefore, it is possible that the high endogenous levels of melatonin in C3H/HeN [40] does not allow to explore the effects of exogenous melatonin on hippocampal neurogenesis as is found in Balb/C- and C57Bl6-mice (i.e., [24,25]).

Melatonin acts through several mechanisms involving the activation of membrane receptors [1,2], as a scavenger of free oxygen radicals, and as a modulator of cytoskeletal rearrangements [12,41,42]. Interestingly, melatonin favors some events in the neuronal development process in the adult dentate gyrus [14,43]. The neurogenic process involves several biological events, including cell proliferation, migration, survival, and differentiation [44].

Moreover, melatonin stimulates dendrite maturation and increases the complexity of newborn neurons after fourteen days of treatment in C57Bl/6 mice [24]. This effect may be related to its capability to modulate microtubule polymerization [11] that is important for both dendrite maturation and axon differentiation [45]. In this sense, it is in the dentate gyrus that newborn neurons send their axons to the CA3 region via the mossy fiber tract to form functional synapses with pyramidal neurons [29]. The axons of newborn neurons also contribute to the formation of the infrapyramidal mossy fiber projections [30]. Here, melatonin increased the volume of the infrapyramidal mossy fiber projection and the volume of the suprapyramidal mossy fiber projection in male Balb/C mice after one- or six-months of treatment. Regarding the melatonin-induced increases in the volumes of mossy fibers subfields here identified by staining with calbindin, the effects of the indole may be related to its capability to activate membrane receptors that underlie increased expression of calbindin in isolated neurons [46]. Thus, the indole might also favor the outgrowth of axons from newborn neurons to contribute to the formation of the infrapyramidal mossy fiber projection, as observed in adult mice exposed to another pro-neurogenic modulator, such as an enriched environment [30]. However, melatonin also increased the volume of the suprapyramidal mossy fiber projections, suggesting that the indole also affects the structural plasticity of axons in mature granule cells in male Balb/C mice. Moreover, melatonin increased the volume of CA3 subfield, suggesting that the indole also promotes the formation of functional synapses with CA3 granule cells [29]. When considering the above, the evidence is suggestive of a possible mechanism by which melatonin increases the volume of mossy fibers and CA3 through the increase expression of calbindin, which is the main calcium-buffering protein in mossy fibers, as a consequence of the activation of the melatonin membrane receptors [31,32,33]. However, this hypothesis must be addressed in a specific and more complex study.

Interestingly, the structural plasticity of mossy fiber projections coincides with better performance on several behavioral tests related to learning and memory [31,32,33]. However, the correlation between the increased neurogenesis and volumes of mossy fiber projections in the dentate gyrus with the antidepressant like effects of melatonin is not known [23,25,47,48,49,50]. Nevertheless, the antidepressant like effects of melatonin might be related to its effects on different stages of neuronal development, including the pro-survival effect, maturation of dendrites, axon outgrowth of newborn neurons, the structural plasticity of axons in mature granule cells, and with specific molecular mechanisms. However, this hypothesis must be addressed in a specific study, which is currently being conducted, in which adult hippocampal neurogenesis is diminished.

Regarding the molecular mechanism involved in the melatonin-induced formation or modification of axons, a recent study provided evidence for the participation of protein kinase B, also known Akt, in the pathway stimulating axonogenesis and synaptic transmission in central neurons [51]. In addition, cytoskeletal rearrangements caused by melatonin may involve the activation or participation of intracellular targets such as PKCα and CaM [3,4,5,6,11,13], which are proteins with the capability to bind calcium via their EF-hand domains. Thus, based on the activation of calcium-dependent proteins, on its effects to regulate the generation of new neurons in mice, and on the expression of calretinin, another calcium-binding protein that is essential for neurogenesis [3,4,5,6,11,13,52,53,54], melatonin is considered as a molecule that connects calcium signaling and neuronal development [55].

Our results are based on the staining pattern for calbindin, a marker expressed by mature granule cells [30]. Calbindin is a calcium-binding protein that functions as a calcium buffer but may also act as a calcium sensor [34]. This protein is important for neuronal survival and is required for plasticity and information processing [56] In this study we confirmed that calbindin, similar to calretinin [15], stained the nucleus within cells with oval and round somas, but the protein was also located in the dendrites of newborn granular cells in the dentate gyrus [16], allowing us to visualize the complete mossy fiber projection [30].

Moreover, melatonin increased the volumes of mossy fiber projection in male Balb/C mice after six-months of treatment. This time point is relevant because during aging melatonin exerted the most significant positive effects on neurogenesis in the dentate gyrus of young mice treated for six months. The generation of new neurons decreases significantly in the dentate gyrus at this time point [24]. Thus, melatonin may promote and maintain neurogenesis and structural plasticity in the axons forming mossy fiber projection in a critical period in which the generation of new neurons decreases in the dentate gyrus of male Balb/C mice [24]. Therefore, exogenous melatonin may be a relevant treatment for some neuropsychiatric disorders and neurodegenerative diseases that progress with a decrease in the plasma levels of melatonin, such as Alzheimer’s disease, other forms of senile dementia, schizophrenia, and obsessive-compulsive disorder. Decreased plasma melatonin levels have also been observed under stressful conditions and during aging [28,57,58,59,60,61,62,63,64,65]. The decrease in plasma melatonin levels may follow the deterioration of the suprachiasmatic nucleus (SCN), which acts as the circadian pacemaker [66,67,68,69,70].

Finally, our study provides support for melatonin as an important endogenous modulatory factor that promotes plasticity, as evidenced by increased volumes of the mossy fiber projection that are formed by axon bundles of newborn and mature neurons in the dentate gyrus. In this case, the effects of melatonin on mossy fibers, as identified by calbindin staining, suggest that the indole is an important modulator of several events of the neuronal development process. In addition, our results for calbindin staining support the hypothesis that melatonin may be a molecule connecting calcium signaling to neuronal development [55].

## 4. Materials and Methods

### 4.1. Animals and Melatonin Treatment

For the analysis of the effects of melatonin on the structural plasticity of mossy fibers, we used material form our previous study [15] in which the handling mice was in accordance with the institutional and legal regulations regarding animal ethics (IACUC SIC092025, 20 September 2011 by the Ethics committee of the National Institute of Psychiatry). Twenty-male Balb/C mice were obtained from Harlan (Tlalpan, Mexico City, México). They were housed in standard laboratory cages under 12-h light/12-h dark cycles at a temperature of 23 ± 1 °C in the animal facilities of the National Institute of Psychiatry “Ramón de la Fuente Muñiz”. Mice were exposed to food and water ad libitum. Mice of 8 weeks old were treated with melatonin or vehicle as control. Melatonin was provided in the drinking water. Following this, the animals were treated with melatonin in concentrations to yield a dose of 8 mg/kg of body weight per day for one or six months. The estimated daily melatonin intake for each mouse was based on average daily water consumption. Thus, the concentration of melatonin was adjusted in accordance to the body weight changes along the time of treatment considering an average daily water consumption rate of 5 mL per day. Melatonin was freshly prepared every third day and dissolved in a minimum volume of ethanol (0.15%) plus tap water and provided in feeding bottles that were protected from light [71]. Mice in the control groups received water containing minimum volume of ethanol plus tap water. The water consumption was similar for all groups. The dose of administered melatonin was chosen considering our previous research on the effects of melatonin on adult hippocampal neurogenesis that had strong effects in C57Bl6 mice [24,52] and in Balb/C mice [24,52].

To study the effects of melatonin on the structural plasticity of mossy fibers, rodents of 8 weeks old were treated during one- or six-months and at the end of treatment, mice were 3 or 8 months old, respectively (Figure 2). The time points included in this study were chosen in accordance with our previous study in which melatonin showed a time-window for causing the most significant positive effects on neurogenesis in the DG in mice of 8 weeks old treated with melatonin during six-months [24].

### 4.2. Tissue Processing, Immunohistochemistry, Total Number of Calbindin-Labeled Cells in the Granular Cell Layer in the Dentate Gyrus, and Morphometric Analysis

Brain coronal sections of 40 µm thick were stained using free-floating immunohistochemistry [72] to determine the volume of mossy fiber projection in the DG in series of every 6th tissue section from the DG in all animals as described elsewhere [23]. Mossy fibers were identified with a rabbit anti-calbindin antibody (1:5000, Swant, Switzerland). Secondary biotinylated anti-rabbit antibody was from Jackson Immunoresearch (West Grove, PA, USA) [23,25]. Mossy fibers were analyzed throughout the rostro-caudal extent of the granule cell layer (GCL).

The total mossy fiber projection volume was estimated with the Cavalieri principle and the absolute number of of calbindin-positive cells in the granular cell layer in the dentate gyrus was determined in coronal 40 µm sections, 240 µm apart, covering the complete dentate gyrus in its rostro-caudal extension [36] in melatonin- or vehicle-treated mice using Image Pro Plus software (Media Cybernetics, Warrendale, PA, USA) driving a motorized stage on a BH2 Olympus microscope with a 4× objective. We also assessed the volume of the hilus, the suprapyramidal- and infrapyramidal-mossy fiber projections, and CA3. Areas sizes of the mossy fibers subfields were determined in 10 to 11 coronal sections per animal containing the DG. To obtain the volume of the mossy fibers subfields, the sum of areas measured was multiplied by the inverse of the sampling fraction (6) and 40 (the section thickness in micrometer) and the total number of calbindin-positive cells (N) was estimated using the following formula: Estimate = *N*/asf × tsf × ssf where *N* is the number of cells counted, “asf” is the area sampling fraction, “tsf” is the thickness sampling fractions and “ssf” stands for the section sampling fraction. Images showing the granule cell layer were captured with a 10× Plan objective (numeric aperture, NA, 0.22) on a DM500 Leica microscope equipped with a video camera ICC50 (Leica, Buffalo Grove, IL, USA). The total area covered was 1.289 × 10^6^ squared μm. Images showing calbindin staining in the DG were converted to 8 bits to adjust brightness and contrast in the ImageJ software (NIH, Bethesda, MD, USA). Following this, granule cells in fields within the granule cell layer were counted within the cell counter plugin and the area of the DG was determined in the ImageJ software.

### 4.3. Immunoblotting

The specificity of the anti-calbindin antibody used to identify mossy fibers was corroborated by western blot [23] using dissected hippocampus of adult mice. Membranes were incubated with rabbit anti-calbindin antibody (1:1000; Swant, Germany). Proteins were visualized with the enhanced chemiluminescence detection system (Millipore, México City, México) in a ChemidocTM touch System (Bio-Rad, México City, México).

### 4.4. Statistics

Analysis was performed using SigmaStat 3.1 software (Systat Software, San Jose, CA, USA). Results are presented as mean ± standard error of the mean. Mean differences between groups were analyzed with a two-way ANOVA followed by Student-Newman Keuls test (factor A: Treatment, factor B: Time). Differences were considered statistically significant at *p* ≤ 0.05.

## Abbreviations

DG	Dentate gyrus
CA	Cornus ammonis
VEH	Vehicle
PKC	Protein kinase C
CaM	Calmodulin
Akt	Protein kinase B
